# Multi-Level Optimization for Data-Driven Camera–LiDAR Calibration in Data Collection Vehicles

**DOI:** 10.3390/s23218889

**Published:** 2023-11-01

**Authors:** Zijie Jiang, Zhongliang Cai, Nian Hui, Bozhao Li

**Affiliations:** School of Resource and Environmental Sciences, Wuhan University, Wuhan 430079, China; jiangzijie@whu.edu.cn (Z.J.); huinian@whu.edu.cn (N.H.); libozhao@whu.edu.cn (B.L.)

**Keywords:** camera–LiDAR calibration, automatic calibration, targetless registration, data fusion, autonomous driving

## Abstract

Accurately calibrating camera–LiDAR systems is crucial for achieving effective data fusion, particularly in data collection vehicles. Data-driven calibration methods have gained prominence over target-based methods due to their superior adaptability to diverse environments. However, current data-driven calibration methods are susceptible to suboptimal initialization parameters, which can significantly impact the accuracy and efficiency of the calibration process. In response to these challenges, this paper proposes a novel general model for the camera–LiDAR calibration that abstracts away the technical details in existing methods, introduces an improved objective function that effectively mitigates the issue of suboptimal parameter initialization, and develops a multi-level parameter optimization algorithm that strikes a balance between accuracy and efficiency during iterative optimization. The experimental results demonstrate that the proposed method effectively mitigates the effects of suboptimal initial calibration parameters, achieving highly accurate and efficient calibration results. The suggested technique exhibits versatility and adaptability to accommodate various sensor configurations, making it a notable advancement in the field of camera–LiDAR calibration, with potential applications in diverse fields including autonomous driving, robotics, and computer vision.

## 1. Introduction

The fusion of LiDAR (Light Detection and Ranging) data and camera image data is an essential step in many fields such as autonomous driving, 3D reconstruction, urban planning, and environmental monitoring [1,2,3]. The significance of data fusion arises from the dissimilarities between LiDAR and camera image data, which vary in terms of spatial resolution, velocity and distance estimation capacities, resistance to adverse weather conditions, and sensor sizes, among other factors [4]. LiDAR sensors are capable of accurately capturing 3D spatial information while lacking color and texture data that images can provide [5,6]. By fusing these two types of data, it is possible to create more accurate and detailed maps of the environment, which is crucial for applications like autonomous driving [7]. What is more, combining LiDAR and image data can improve the detection and recognition of objects in a scene. LiDAR can detect the presence and position of objects while images can provide details about their appearance and texture. The fusion of these data sources can lead to better object detection and tracking, which is vital for applications such as robotics and autonomous vehicles. Lastly, LiDAR is capable of measuring distances with high accuracy but it cannot provide depth perception of objects that are hidden from view. Combining LiDAR and image data can help overcome this limitation by providing a more comprehensive understanding of the scene’s depth and structure. Overall, the fusion of LiDAR and image data provides a more comprehensive understanding of the environment, which is essential for a wide range of applications. It enables improved accuracy [8], object detection [9,10], depth perception [11,12,13], and 3D modeling [14,15], making it an essential technique for many fields.

When it comes to the fusion between LiDAR and image data, the precise calibration between the two sensors plays a key role [16,17,18]. LiDAR sensors capture 3D point clouds of the environment, and camera sensors capture 2D images. To create a comprehensive 3D model, it is necessary to transform the camera image data into 3D coordinates that match the LiDAR point cloud data. Calibration ensures that the transformation is accurate, which is essential for generating accurate point clouds. Accurate calibration of LiDAR and camera sensors also enables better object detection and tracking [9,10]. By precisely aligning the two sensor data streams, it is possible to accurately locate objects in 3D space. What is more, calibration reduces measurement errors and noise, which can improve the overall accuracy of the data fusion process. This is particularly important for LiDAR data, which can be affected by noise caused by reflections and other factors.

Precise calibration is essential for achieving accurate data fusion between LiDAR and camera sensors. However, calibration is not a one-time process as the sensors may shift and change over time. This is particularly prevalent in data collection vehicles where the sensors are affixed to a mobile vehicle, resulting in a greater likelihood of modification to their relative pose. It will be much more time-consuming and resource-intensive if the data collection vehicle has to be sent back to the calibration field to do a thorough calibration. Accordingly, the implementation of data-driven precise calibration serves as a valuable means to account for such fluctuations [19], where the calibration parameters are updated using the data acquired by LiDAR and camera sensors. By updating the calibration parameters as necessary, data-driven calibration can adjust for changes in the environment and improve the accuracy of the data fusion process without the need for extensive calibration in a dedicated calibration field. This agile calibration approach saves time and resources while maintaining the integrity of the calibration process.

Currently, a considerable body of research is dedicated to the calibration of LiDAR and camera systems. Some approaches, as described in [20,21,22], rely on predefined targets that are visible in both the camera and LiDAR data to estimate calibration parameters. However, to eliminate the need for pre-deployed targets, several calibration methods leverage feature extraction and matching techniques. These methods utilize various types of features, including point features [23,24,25], line features [26,27,28], surface features [29], semantic features [30], and 3D structure features [31]. Instead of establishing explicit feature correspondence between the camera image and the point cloud, certain methods [19,32,33,34,35,36,37,38] employ general appearance similarity as a metric to evaluate calibration quality, formulating the calibration problem as a nonlinear optimization task. Additionally, a few alternative approaches [39,40,41,42,43,44,45,46] based on deep learning have emerged as promising paradigms for addressing this calibration challenge.

Despite the variety of approaches employed, a general framework for gaining insight into the calibration process is still needed. Additionally, current methods tend to be highly sensitive to the initial calibration parameters. Thus, poor initial calibration parameters can lead to suboptimal calibration results using these approaches. Therefore, the primary objective of this research paper is to propose a novel and practical optimization method for the data-driven calibration of camera–LiDAR systems in data collection vehicles. Specifically, this approach aims to address the challenges associated with poor initial calibration parameters.

The paper presents several contributions:

(1) A comprehensive and general model is proposed for the camera–LiDAR calibration;
(2) An objective function that takes poor calibration parameters into account is introduced;
(3) A multi-level optimization technique that achieves both calibration precision and computational efficiency is developed.

These contributions enhance the state-of-the-art in camera–LiDAR calibration and provide valuable insights for future research in this field.

In the subsequent sections of this paper, we present a comprehensive discussion of the related work, proposed methodology, experimental results, and conclusion. Specifically, Section 2 delves into the relevant literature and prior research in the domain of camera–LiDAR calibration. Section 3 delineates the proposed method, which is divided into several subsections for clarity. Section 3.1 offers a high-level overview of the devised model, which aims to achieve data-driven calibration between a camera and a LiDAR system. In Section 3.2, we elucidate the data encoding technique employed to extract edge features from dense point clouds and camera images, with an emphasis on its specific characteristics. Subsequently, Section 3.3 presents the proposed objective function, which serves as a metric for evaluating the alignment between the camera and LiDAR data. In Section 3.4, we expound on the multi-level optimization process, which refines the calibration parameters in a systematic manner. Following the detailed explanation of the proposed method, Section 4 showcases the experimental results and engages in pertinent discussions to highlight the efficacy of the approach. Finally, Section 5 provides a conclusive summary of the paper and outlines potential avenues for future research.

## 2. Related Work

Research on the calibration problem between LiDAR and cameras has been ongoing since LiDAR began to be used in vehicles. Calibration techniques can be broadly classified as either offline or online. Offline methods require a predefined target and are typically carried out in an offline setting. Online methods, on the other hand, rely on LiDAR and camera data and are more suitable for on-road applications. Online methods can be further classified into feature matching-based and statistical-based approaches. Statistical-based methods, also known as direct methods, use all the available information without finding corresponding points. Feature-based methods, also known as indirect methods, involve finding the corresponding points and utilizing that information. Features may include points, lines, or surfaces. In recent years, several calibration approaches utilizing deep learning techniques have also emerged. In general, calibration approaches can be classified into four categories: target-based approaches, feature matching-based approaches, statistics-based approaches, and deep learning-based approaches, as shown in Table 1.

### 2.1. Target-Based Approaches

Target-based approaches for camera–LiDAR calibration rely on a predefined target that is visible by both sensors. Typically, the target is designed to have a known geometric structure and can be represented in both sensor data, albeit in different forms.

The offline calibration method, using a calibration board as described in [20], can accurately calculate the relative pose between a laser rangefinder and a camera by placing the calibration board indoors. However, this method cannot be performed in real-time as the relative pose between the laser rangefinder and camera is constantly changing during vehicle operation, rendering this method ineffective. Similarly, the method of using pre-positioned ground control points for the registration of unmanned aerial vehicle images and onboard laser point clouds, as described in [21], also faces this problem.

### 2.2. Feature Matching-Based Approaches

Feature matching-based approaches typically involve first converting the LiDAR and camera data into a common coordinate system using the initial calibration parameters. Next, salient features are extracted from the LiDAR and camera data, such as corners or edges, using feature detection algorithms. These features are then matched between the LiDAR and camera data based on their descriptors, which are high-dimensional representations of the features. Once the matching features are identified, the calibration parameters can be estimated using optimization methods, such as the PnP algorithm or bundle adjustment. These methods compute the transformation between the LiDAR and camera coordinate systems that minimize the reprojection error between the matched features.

A feature-based method that uses Harris corner points of road markings for matching is described in [23]. This method projects the point cloud onto a plane to form an intensity image, which is then matched with the image data. Similarly, [24] extracts Harris corner points from images and performs an exhaustive search for corresponding points in the LiDAR data, with the use of the Fourier transform for computational acceleration. In [25], the authors utilize SIFT [49] to extract intensity features from point cloud images for point cloud registration.

In [26], skyline features are extracted from both the point cloud projection and the image, and an ICP [50] algorithm considering the point normal vectors is used to find the corresponding points on the skyline. Finally, the camera pose is calculated based on the corresponding points. Similarly, [27] uses a brute-force search to iteratively solve the registration parameters, and the search range is reduced by half after each iteration. Furthermore, [28] is also based on line matching. Canny edge lines are extracted from both the image and the point cloud projection, and the camera pose is calculated based on the correspondence relationship between the lines using the generalized collinearity equation.

The method proposed by [29] is based on surface matching. The method involves extracting features from both the point cloud and the digital image, then using a feature descriptor to match corresponding features. The matching is performed on planar surfaces, and the camera pose is estimated using an iterative closest point algorithm. This method can achieve high accuracy but it relies on the availability of planar surfaces in the scene.

Ref. [30] proposed a camera–LiDAR calibration method based on semantic segmentation of images. Specifically, they extracted feature objects through the semantic segmentation of images and constructed a cost function based on the matching degree of the LiDAR points projected into the feature object region. The proposed method utilized semantic information, which is a higher-level representation, and thus demonstrated robustness to scene noise compared to edge-based methods. However, this method requires specific scene requirements, such as recognizable objects with certain shapes like cars, which limits its applicability in mapping applications.

In [31], sparse point clouds are constructed through structure from motion (SFM) [51] from images, and rigid ICP is used to align the sparse point clouds with the LiDAR point clouds. However, this method is essentially an offline method since it mainly uses continuous image frames to construct sparse point clouds, and then performs ICP alignment and the joint BA solution with the LiDAR point clouds.

### 2.3. Statistics-Based Approaches

Statistics-based approaches typically involve projecting LiDAR point clouds onto the camera image plane using the initial calibration parameters. This creates a 2D projection image that can be compared to the actual camera image. To compare the projection image and the camera image, filtering methods are employed to process the two images separately. These methods may include edge detection, noise reduction, or other image processing techniques. After filtering, the two images are overlapped, and specific statistical measures, such as correlation coefficients or mutual information, can be calculated to measure the similarity between the two images. Once the similarity measures are computed, non-linear optimization techniques can be used to refine the calibration parameters. These techniques aim to minimize the difference between the projection image and the camera image by adjusting the calibration parameters. This optimization process can be iterative, with the calibration parameters updated after each iteration until convergence is reached.

Ref. [32] utilizes the mutual information between image pixel values and laser reflectance intensity, and ref. [33] computes the mutual information between the image pixel values and both the reflectance and the depth maps from the LiDAR data. One drawback of mutual information methods is that they heavily rely on local features, which results in a significant dependence on the initial registration parameters. Moreover, using reflectance values for the mutual information method has a drawback in that it requires calibration of the laser reflectance values, as uncalibrated reflectance values are considered invalid, which can lead to inaccurate similarity measurements. In their work, ref. [19] proposed an approach that extracts edge points from both the camera images and the LiDAR data. The method utilizes an objective function that integrates the information of camera intensity and depth discontinuity in a product sum fashion. It can detect and correct miscalibration between the two data sources through a grid search optimization.

### 2.4. Deep Learning-Based Approaches

Deep learning-based approaches have emerged as a promising method for LiDAR–camera calibration. These approaches aim to replace the manual feature extraction step with neural networks to better handle the complex data involved in LiDAR–camera calibration. By leveraging the powerful representation learning capabilities of neural networks, these approaches can automatically extract features that are more relevant to the calibration task. Moreover, the subsequent feature matching and parameter calculation process can also be implemented using neural networks. This enables the entire calibration process to be performed in an end-to-end fashion, with the neural network taking raw data as input and directly outputting the calibrated parameters.

In a recent study by [39], an end-to-end approach is proposed to tackle the calibration problem. This approach employs convolutional neural networks (CNNs) to extract feature information from both camera and LiDAR-projected images, and subsequently, another CNN block is utilized to establish correspondence between the features. Finally, a fully connected network is employed to output the calibration parameters. Subsequent research [40,41,42,43] has also employed neural networks as a tool to tackle the problem of calibration. Despite their impressive performance in various applications, deep learning models are known to suffer from limitations when it comes to applying them to arbitrary configurations. In such cases, conventional calibration techniques may be more practical and efficient than re-training the models. Moreover, the lack of interpretability of deep learning models makes it difficult to perform failure case analysis and estimate the operational limits analytically, which poses a significant challenge for these black box approaches.

## 3. Materials and Methods

This paper proposes a novel and comprehensive general framework for the online calibration of LiDAR and camera data, which abstracts out the technical details of different methods currently adopted. It also contains specific implementations used for data-driven calibration of dense LiDAR data and camera images in data collection vehicles, which includes a data encoding process that can effectively extract informative and discriminative features from dense LiDAR and camera data, an objective function that takes very poor calibration parameters into account, and a multi-level optimization process that can perform well with poor initial calibration parameters and provide rapid optimization speed. The proposed method addresses several challenges associated with data-driven calibration, especially the requirement for robustness against initial calibration parameter inaccuracies.

**Figure 1:**
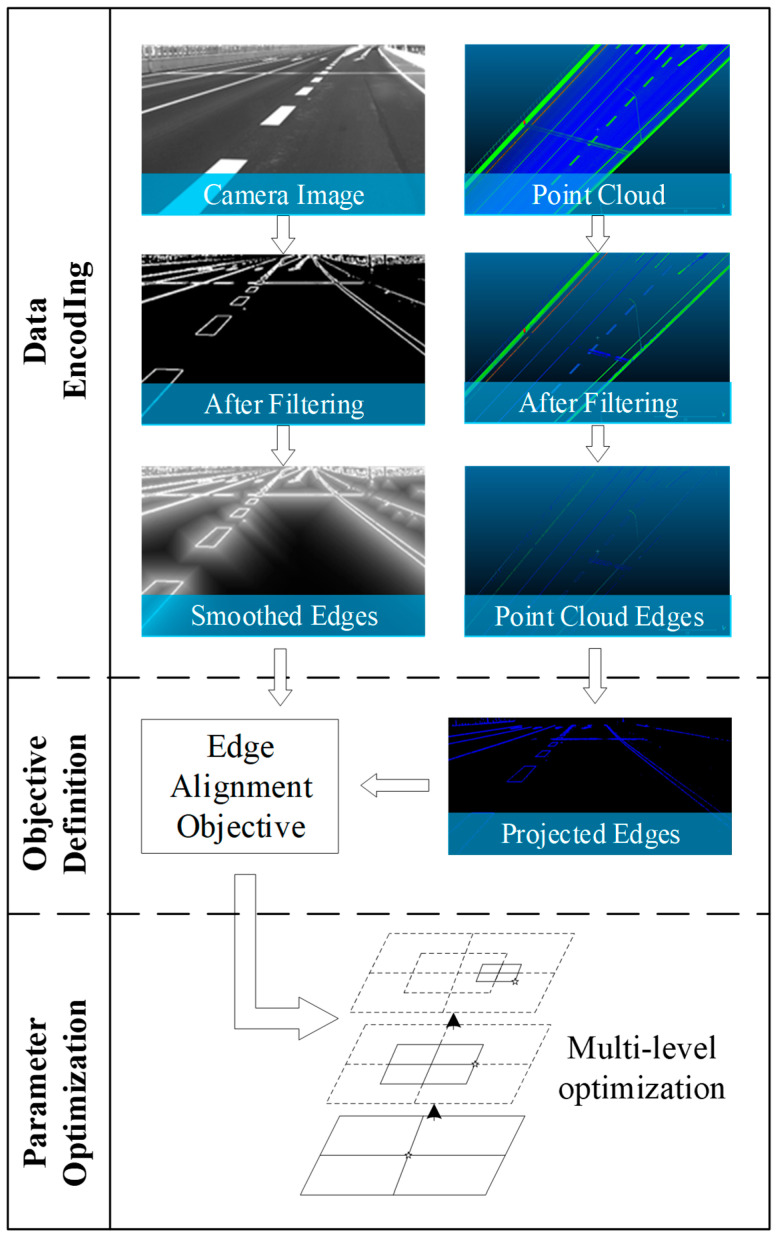
The overview of the proposed data-driven camera–LiDAR calibration method. The camera image captured originally is in size 1920 × 1200, and is cropped to 730 × 442 for better display. The point cloud is viewed in CloudCompare v2.11.3 [52] software and zoomed to the corresponding area for easier comparison. The image titled “Projected Edges” is obtained by projecting the point cloud in “Point Cloud Edges” onto the camera image coordinate system.

The overview of the proposed method is shown in Figure 1.

### 3.1. The Camera–LiDAR Calibration General Model

As discussed above, numerous approaches were developed for online calibration of LiDAR and camera data. However, these methods lack a unified model that can effectively summarize the common techniques utilized. Therefore, there is a need to develop a general model that can integrate the various approaches and provide a comprehensive overview of the online calibration process. Such a model can enable researchers and practitioners to understand the underlying principles and mechanisms of online calibration and provide them with a systematic and efficient approach to achieve optimal results. This model can also facilitate the development of new techniques and methods that can further improve the accuracy and reliability of LiDAR–camera calibration.

The proposed model for LiDAR–camera calibration is composed of three essential stages: data encoding, objective definition, and parameter optimization.

The data encoding stage is responsible for extracting informative and discriminative features from both LiDAR and camera data, which are then utilized for the subsequent calibration process. In feature-based methods, the data encoding pertains to the explicit feature extractor, which may include corner point extractor, skyline extractor, and other similar techniques. In statistical-based methods, the data encoding pertains to the specific statistics utilized in the encoding process. In Equation (1), F stands for the encoding function, its input includes camera image data C, LiDAR data L, and calibration parameters P. It outputs the encoded representation of camera data ${Enc}_{C}$ and LiDAR data ${Enc}_{L}$:

(1) $${Enc}_{C},{Enc}_{L}=F(C,L,P)$$

The objective definition stage defines the objective function to optimize during the calibration process. This objective function seeks to minimize the difference between the measurements obtained from the two sensors, with the ultimate goal of achieving precise calibration between them. The objective function can be designed based on various metrics, such as the mean squared error or the mutual information between the two sensor measurements. In Equation (2), G stands for the objective function and Obj stands for the resulting objective score:

(2) $$Obj=G({Enc}_{C},{Enc}_{L})$$

Finally, the parameter optimization stage performs the actual optimization process, whereby the parameters that maximize the objective function are iteratively adjusted. This process can be carried out using various optimization algorithms, such as gradient descent [53], the Levenberg–Marquardt algorithm [54], etc. The choice of optimization algorithm can depend on the nature of the criterion and the specific requirements of the calibration task. In Equation (3), $P^{*}$ stands for the resulting optimal calibration parameters:

(3) $$P^{*}=argmax(Obj)$$

Putting them together, the camera–LiDAR calibration general model can be expressed as Equation (4):

(4) $$P^{*}=argmax(G(F(C,\;L,\;P)))$$

A synopsis of the representative prevailing literature utilizing the proposed general model is presented in Table 2 to facilitate a better understanding of the overall model.

Overall, the proposed model for LiDAR–camera calibration provides a systematic approach for achieving precise calibration between these two sensors. This model not only facilitates comprehension of existing methods but also establishes a paradigm for the development of future approaches.

### 3.2. Data Encoding in Road Scenes

The purpose of data encoding is to extract informative and discriminative features from the data collected by LiDAR and camera sensors.

Within road scenes, notable visual features, including lane markings and traffic signs, are captured by cameras and also have corresponding representations in LiDAR point clouds. The edges identified from camera images and point clouds demonstrate a significant level of correlation. Consequently, for effective information encoding, it is recommended to extract edges from both modalities. Specifically, in the case of camera images, edge detection should be based on pixel values, while in the case of point clouds, edge extraction should rely on point intensities.

The following details outline the specific implementation approach for the data encoding, designed for both camera image and point cloud modalities.

Image data encoding is divided into two steps:

(1) Edge filtering

To align the image with the point cloud, edge information is used as a reference, therefore here we perform edge filtering on the image to extract the edge information in the image. In simple terms, after converting the original image to grayscale, each pixel value is set to the maximum difference between the original grayscale value of that point and the grayscale values of its eight neighboring points. The image after edge filtering is referred to as E (it should be noted that other filtering methods can also be used for edge filtering here).

(2) Inverse distance transform

After the edge filtering process, an inverse distance transform is applied to smooth the edge image. This helps to make the final objective function smoother and rewards transformations that are closer to the true value, while avoiding getting stuck in local optima. The purpose of this step is to spatially smooth out the distribution of edge information, which in turn helps to make the objective function smoother and ensures a more stable optimization process. The specific approach is described by Equation (5).

(5) $$D_{i,j}=\alpha \cdot E_{i,j}+(1-\alpha )\cdot \underset{x,y}{\max}E_{x,y}\cdot \gamma^{max(\left|x-i\right|,\left|y-j\right|)}$$

Equation (5) employs L1 distance as a computational expedient, allowing for a linear-time application of the transformation with respect to the number of pixels. Consequently, the neighboring pixels are subject to an exponential spillover effect from each edge. The weighing factor, denoted by the symbol α, determines the relative contribution of the central pixel value and its surrounding pixels to the calculation of the resultant smoothed pixel value. Specifically, we set $\alpha =\frac{1}{3}$ and γ=0.98, consistent with the values used in [19].

**Figure 2:**
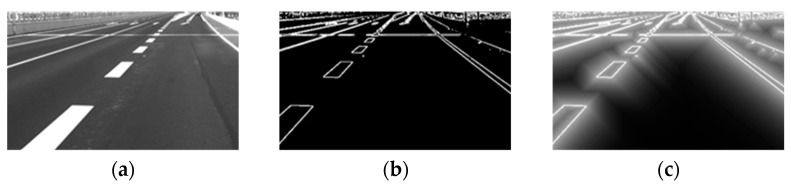
Camera image encoding process. The camera image captured originally is in size 1920 × 1200, and is cropped to 730 × 442 for better display. (**a**) Original camera image; (**b**) image after edge filtering; (**c**) image after inverse distance transform.

Figure 2 shows the results of the camera image encoding process, encompassing the transformation from the original camera image to the resulting smoothed edge image.

**Figure 3:**
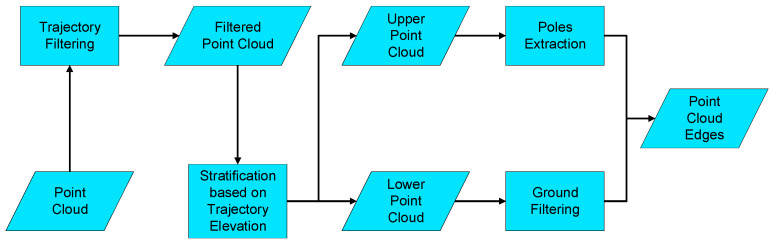
Point cloud encoding diagram.

In addition to camera image data encoding, the encoding of point clouds also holds significant importance. A thorough analysis of point clouds in road scenes reveals numerous discernible features, including lane markings, guardrails, poles, traffic signs, etc. Besides that, it should also be noted that the camera–LiDAR calibration requires precise geometric accuracy in both the longitudinal and the latitudinal directions. Longitudinal direction refers to the direction along the road, and latitudinal direction refers to the direction across the road. When considering the latitudinal geometric accuracy, it is noteworthy that line features on the road, such as lane markings, guardrails, and curbs, are well distributed along the latitudinal direction, rendering them effective for controlling latitudinal accuracy. As for the longitudinal direction, vertical features such as poles and traffic signs have a significant impact on controlling longitudinal accuracy due to their clear geometric contours. However, such features are relatively sparse and susceptible to occlusion and incomplete scanning in real-world scenarios, making them suitable only as supplementary features to play an auxiliary role. What is more, the line features on the road and the vertical features also exhibit notable edges in the camera image. Given these considerations, the utilization of both line features on the road and vertical features appears to be a judicious option. The overall process is illustrated in Figure 3.

(1) Trajectory Filtering

To ensure the accuracy and reliability of calibration and eliminate potential noise in point cloud data, it is often necessary to filter out points that lie outside of the road range. One effective method involves shifting each trajectory point to the left by approximately half of the road’s width, thus generating a series of left boundary points which, when connected, define the left road boundary. Similarly, shifting each point to the right by half of the road’s width produces a set of right boundary points that define the right road boundary. Connecting these left and right boundaries generates a road surface, which can be used to filter the point cloud data and remove any points that lie outside of the defined road range.

(2) Stratification based on Trajectory Elevation

To facilitate further analyses, the collected data points are segregated into distinct upper and lower layers based on the elevation of the corresponding trajectory points.

(3) Ground Filtering

The process of extracting the road surface from the lower layer data involves the application of several techniques. First, the CSF filter is employed to identify and isolate the relevant data points on the road. Next, the Otsu threshold method is utilized to perform background removal, which separates the points of interest from background points on the road surface. The efficacy of this approach can be attributed to the material differences between the points of interest and background points, which result in a large discrepancy in reflection intensity. By effectively utilizing thresholding techniques, the relevant data points can be accurately extracted while minimizing the impact of background noise. Once the data are appropriately thresholded, linear feature edges such as lane lines, guardrails, and curbstones can be extracted easily.

The CSF filter [56] and Otsu [57] thresholding techniques are combined to effectively isolate the relevant data points and remove extraneous background noise. Following this, the normal vectors of the point cloud are extracted and carefully analyzed to determine whether each point falls on an edge, based on the angle between the normal vectors.

(4) Poles Extraction

To analyze the upper layer data, the principal component analysis (PCA) [58] technique is utilized to extract point cloud clusters along the z-axis. This process is effective in identifying and isolating vertical elements such as poles and traffic signs. PCA is a statistical approach that identifies the major axis of variance in a given dataset, and the associated eigenvectors can be utilized to determine the orientation of the object under consideration. By analyzing the orientation of the eigenvectors associated with a point cloud, vertical objects can be distinguished from the surrounding environment. The edge of the vertical objects can then be extracted based on the normal vectors calculated from the point cloud.

**Figure 4:**
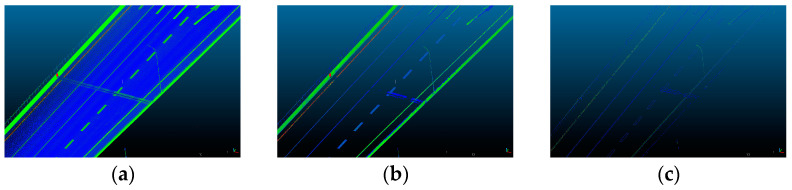
Point cloud encoding process. The point cloud is viewed in CloudCompare [52] software and is zoomed in to achieve an almost bird’s-eye viewpoint, providing a clearer and more detailed representation. (**a**) Raw point cloud; (**b**) point cloud after filtering; (**c**) point cloud after edge extraction.

The point cloud under different stages is demonstrated in Figure 4.

### 3.3. Enhanced Objective Definition via Suppression of Identical Pixel Contributions

The edges extracted from the camera image and the dense point cloud are two different representations of the same edge in the real world. If the calibration parameters are accurate enough, it is reasonable to assume that the two edges will perfectly overlap with each other. Based on this assumption, the basic idea of our camera–LiDAR calibration algorithm is to define an objective function to describe the degree of overlap between the edges in the image and the edges in the point cloud, and then search for the optimal calibration parameters that maximize the value of the objective function.

**Figure 5:**
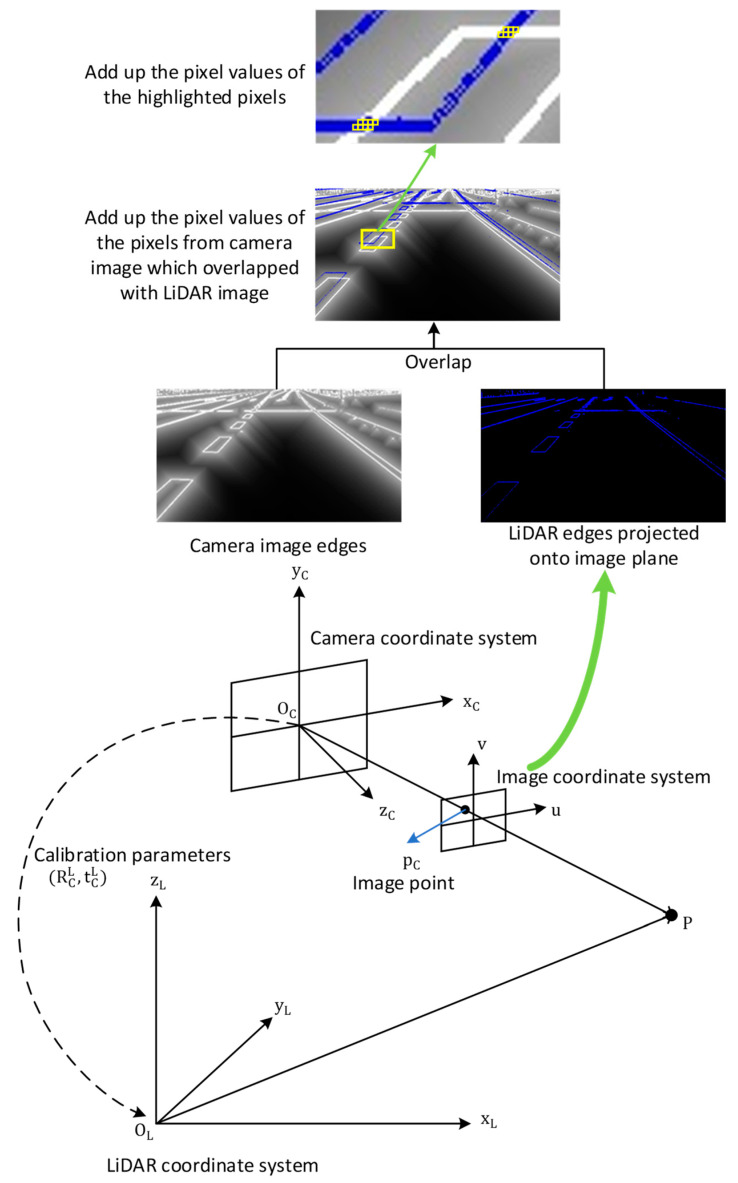
Objective function definition. The LiDAR edge image was generated by projecting the edges of the point cloud onto the coordinate system of the camera image. This projection can be understood as visualizing the point cloud from the perspective of the camera’s optical center.

The calibration parameters are expressed as a rigid body transformation consisting of six degrees of freedom. The rotational component is represented by three Euler angles, while the translational component is represented by three lengths. This spatial transformation enables the precise alignment and positioning of objects in three-dimensional space. To obtain the corresponding objective function value $O^{C}$ for a given set of calibration parameters C, we can project the point cloud $R^{i}$ onto the image plane $D^{i}$ using the parameters C; then, we can calculate the objective function value by summing the pixel values of the corresponding image edges overlapped with the projected point cloud edges. Here, we do not sum up the product of the camera pixel value and the corresponding pixel value from point cloud edges projection. That is because pixel value from point cloud edge projection does not represent the real edginess of the point cloud. Therefore, simply summing up all the camera image pixel values overlapped with the projected point cloud edges is enough and reasonable. For multiple frames, we simply sum up the results obtained for each frame. This process is illustrated in Figure 5.

The objective function can be expressed mathematically using Equation (6).

(6) $$O_{C}=\sum_{f=n-w}^{n}\sum_{p=1}^{\left|R^{f}\right|}D_{i,j}^{f}$$

In Equation (6), *f* denotes the frame number and *w* represents the data window size used for calculating the objective function value. The outermost sum operator means the sum of multiple frames. For example, w=1 means only considering the most recent frame. *p* denotes the index of a point in the point cloud data, while i and j represent the image coordinates corresponding to the point after projection onto the image plane from the point cloud.

Despite the plausibility and potential effectiveness of the objective function, an unusual phenomenon was observed whereby, under highly suboptimal calibration parameters, the objective function attains an unusually large value. This has the potential to cause the subsequent optimization process to converge towards the suboptimal calibration parameter. Upon investigation, it was discovered that this occurrence was due to a substantial proportion of points within the point cloud being projected onto identical pixels on the image plane, specifically the edges of the camera image. This, in turn, resulted in the objective function acquiring an exceedingly undesired high value.

To avoid situations where multiple points in the point cloud are projected onto a single pixel with a high pixel value, a mechanism was introduced where a pixel only contributes to the objective function once, which will be referred to as the suppression of identical pixel contributions. In other words, if a pixel already has a point cloud projection, subsequent projections of the point cloud onto that pixel will not contribute to the objective function anymore. During the optimization process, there may be cases where incorrect calibration parameters cause the point cloud to project onto the image in a cluster of points, resulting in a significantly large objective function value. However, this calibration is obviously incorrect. Therefore, to avoid such abnormally large objective function values, we suppress the contributions of duplicate projections onto the same pixel when calculating the objective function. The suppression of identical pixel contributions can be seen in the pseudocode of Algorithm 1.
**Algorithm 1:** Pseudocode of objective function definition**Input:** Point cloud edges data, camera image edges data, calibration parameters. **Output:** Objective function value. 1. Initialize a pixel coordinates pool Pool to store the pixel coordinates that have already contributed in the objective function calculation, initialize the objective function value as Obj. 2. Use the calibration parameters to project the point cloud edges data onto the camera image plane, which results in a list containing the corresponding pixel coordinates ${List}_{P}$. for pixel (x,y) in ${List}_{P}$ do 3. if (x,y) not in Pool then Add the pixel value with coordinate (x,y) in the camera image edges data $I_{c}\left(x,y\right)$ to the objective function value Obj. Add the coordinate (x,y) into the pool Pool. 4. Return the objective function value Obj.

### 3.4. Multi-Level Parameter Optimization

Prior research has demonstrated that the objective function resulting from these detailed textures exhibits prominent non-convex characteristics. Consequently, the impractical approach would entail conducting a comprehensive exploration of the calibration parameters to identify the parameter combination that maximizes the objective function. However, a global search of the parameters is time-consuming and unrealistic. A feasible method is to utilize the property of the objective function having a locally convex nature and perform an iterative local grid search optimization of the parameters. The search process for optimizing calibration parameters typically involves iteratively perturbing each parameter by a small increment, referred to as a step size, in order to explore the parameter space and identify the optimal parameters. If a search process takes r steps in one search round, the search space for one parameter will encompass (2r+1) candidate values, where r is referred to as the search radius henceforth. Considering we have 6 parameters in total to perturb, there will be ${\left(2r+1\right)}^{6}$ parameter combinations in the whole parameter search space. During one round of search, the objective value is calculated for each parameter candidate in the search space. After finding out the maximum objective value, the corresponding parameter will be used as the initial parameter for the next search round. The iterative process will continue until the parameter value reaches a point of convergence, defined as the maximum objective value no longer exhibiting significant changes. The computational burden of a single round of iterative grid search can be quantified by Equation (7).

(7) $$L=C{\left(2r+1\right)}^{6}$$

The equation incorporates a factor denoted as C, which accounts for the iterative nature of the process. This factor may be considered constant, as it is not dependent upon the method proposed in this paper.

In fact, the parameter search space is a continuous space. And in practice, the continuous search space is discretized to make the search process feasible. Two important concepts in this discretization process are the search step size and the search radius. The search step size is the discretization unit and determines the discretization precision and hence the optimization granularity. If the search step size is set relatively large, the final optimization result may not be precise enough and deviate more or less from the theoretical optimal value. On the other hand, the search radius is the steps taken in each search round. The product of the search step size and the search radius is called the parameter search range. The parameter search range determines the “field of view” for each iteration during the optimization process, which determines the sensitivity of the optimization process to local non-convexity and hence the robustness of the optimization result.

The parameter search space is typically continuous, but in practice, it is discretized to enable feasible search processes. Two important concepts in this discretization process are the search step size and the search radius. The search step size determines the discretization precision and optimization granularity, and if set too large, may result in imprecise optimization results. In contrast, the search radius is the steps taken in each search round. The product of the search step size and the search radius is called the parameter search range, which determines the “field of view” for each iteration during the optimization process. This field of view affects the sensitivity of the optimization process to local non-convexity and hence the robustness of the optimization result.

Both the search range and search step size are important for the exploration capabilities of a search process. A larger search range can help prevent the search process from getting stuck in a local maximum, while a smaller search step size ensures that the search space is not discretized too coarsely, thus avoiding the oversight of many optimal results. However, if the search range is fixed, a smaller search step size will result in a larger search radius, which can exponentially increase the computational burden of the search process, as discussed previously.

**Figure 6:**
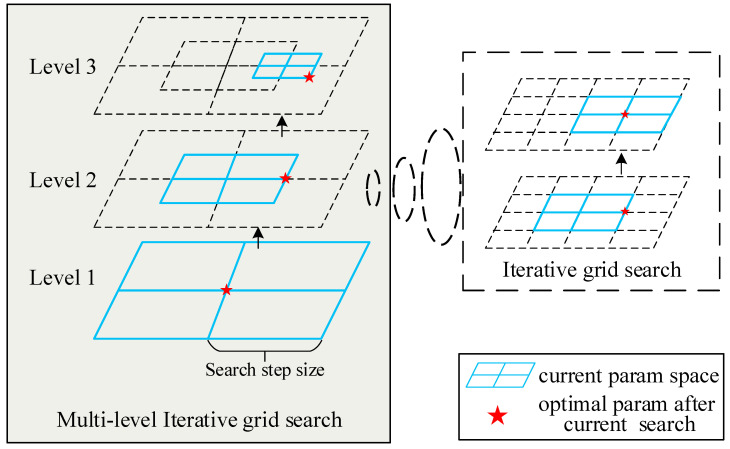
Multi-level optimization process using search radius 1.

To address this quandary, a multi-level optimization approach is proposed, inspired by the image pyramid model. This approach employs a coarse-to-fine strategy, beginning with a large search step size and a small search radius for the initial iterative grid search. This combination provides a large search range but with a relatively small search radius. The resultant parameters are then used as initial parameters for the next level of iterative grid search, where the search step size is decreased to a smaller value while the search radius remains unchanged. This updated configuration is used to conduct the new level of iterative grid search. The search process continues in this manner until the search step size is small enough to indicate that the result is precise enough. Figure 6 provides a visual illustration of the entire process.

Assuming a desired search range of R and a desired search step size of $\tilde{s}$, in the conventional single-level case, the search radius $r_{s}$ should satisfy condition (8):

(8) $$\frac{R}{r_{s}}<\tilde{s}$$

Using condition (8), we can derive the following Equation (9), given that the search radius $r_{s}$ should be an integer:

(9) $$r_{s}=\left\lceil \frac{R}{\tilde{s}}\right\rceil$$

By applying Equation (7), we can derive the following Equation (10) for the computational burden of a single-level search:

(10) $$L_{s}={C(2\left\lceil \frac{R}{\tilde{s}}\right\rceil +1)}^{6}$$

For the multi-level search method, assuming a search radius of $r_{0}$, the initial search step size should be set as $\frac{R}{r_{0}}$. After each level, the search step size is divided by a constant K, resulting in a search step size of $\frac{R}{r_{0}K^{l}}$ after l levels. The search process terminates when the search step size is smaller than the desired search step size of $\tilde{s}$. Therefore, we can derive condition (11):

(11) $$\frac{R}{r_{0}K^{l}}<\tilde{s}$$

Using condition (11), we can derive the following Equation (12), given that the level number l should be an integer:

(12) $$l=\left\lceil {log}_{K}\frac{R}{r_{0}\tilde{s}}\right\rceil$$

By applying Equation (7), we can derive the following Equation (13) for the computational burden of a multi-level search:

(13) $$L_{m}={C(2r_{0}+1)}^{6}\left\lceil {log}_{K}\frac{R}{r_{0}\tilde{s}}\right\rceil$$

By comparing Equations (10) and (13), we can observe that the computational burden of the single-level search method $L_{s}$ increases exponentially with the desired search range R, while the computation burden of the multi-level search method $L_{m}$ increases at a much slower rate due to the logarithmic effect. In scenarios where the initial calibration parameters exhibit significant deviations from the optimal values, a broader search range may be necessary, thereby favoring the adoption of a multi-level search method over a single-level search method.

## 4. Results and Discussion

### 4.1. Experimental Platform

To validate the efficacy of the proposed approach in this study, a data collection vehicle was utilized, which was equipped with a Z+F PROFILER 9012 2D LiDAR (Zoller & Fröhlich GmbH, Wangen im Allgäu, Germany, 360° field-of-view, scan rate of more than 1 million points per second, maximum scanning speed of 200 profiles/s, maximum range of 119 m) for the acquisition of high-density point cloud data, GPS/IMU devices (Northrop Grumman Corporation, West Falls Church, VA, USA) for the fusion and rectification of the data obtained from the single-beam LiDAR, as well as a Point Gray industrial camera (Teledyne FLIR LLC, Wilsonville, OR, USA) for image data acquisition. The sensors were mounted on a Nissan Qashqai vehicle (Dongfeng Motor Company Limited, Wuhan, Hubei, China) for data collection purposes. In particular, a metal frame was securely mounted on top of the vehicle to hold the 2D LiDAR and camera. The camera was positioned in a forward-facing direction, while the LiDAR was tilted at a vertical angle of 45° for enhanced environmental scanning capabilities.

The acquired dataset comprises 100 Individual frames of data, wherein each frame comprises a camera image and its corresponding LiDAR point cloud. The data were captured along a 5 km stretch of a highway road located in Zhejiang, China. Under sunny weather conditions with a temperature of approximately 15 °C and no significant wind, the data collection vehicle maintained a consistent cruising speed of approximately 20 km/h throughout the data collection process.

The calibration parameters for the alignment of the image and point cloud are initially determined by utilizing the calibration parameters provided by the vehicle manufacturer. To obtain the ground truth calibration parameters, a manual point selection process is employed, wherein ten corresponding point pairs are identified in each frame. Subsequently, the PnP method from the OpenCV library is applied using these point pairs to determine the ground truth calibration parameters with greater precision and accuracy.

Our algorithm is implemented in C++, and the experimental results described below were obtained on a laptop computer with an Intel i7-10875H CPU (Intel Corporation, Santa Clara, CA, USA), 16 GB RAM, and a Nvidia GeForce RTX 2080 Super with Max-Q Design GPU (Nvidia Corporation, Santa Clara, CA, USA). The objective function calculation was accelerated using the CUDA framework for GPU computation.

### 4.2. Evaluation and Discussion

A crucial factor affecting the optimization process is the properties of the objective function. Therefore, it is imperative to undertake a thorough analysis of the proposed objective function’s properties.

**Figure 7:**
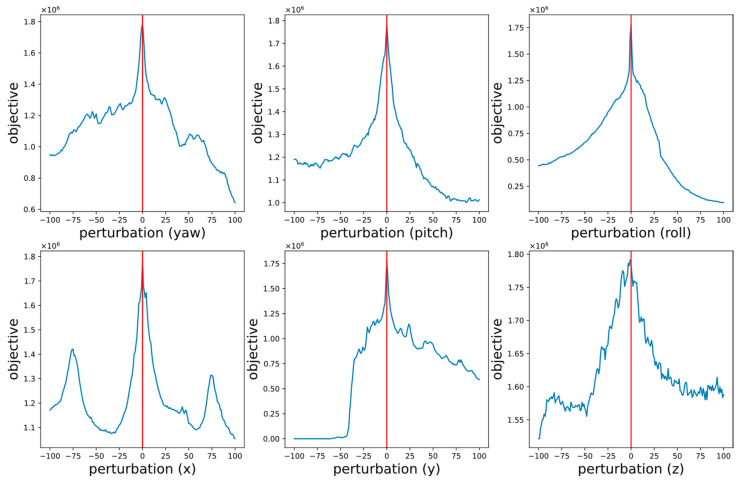
Distribution of the proposed objective function.

Figure 7 shows the distributions of the objective function values obtained by perturbing the six parameters on the basis of a pre-registered parameter. The distributions correspond to the perturbations of yaw, pitch, roll, and x, y, z, respectively. The perturbation unit is (0.125°, 5 cm).

The depicted figure displays that the optimal value of the objective function is attained at the actual value (located at 0 on the abscissa), indicating that maximizing the objective function would lead to obtaining the correct calibration parameters. Furthermore, the function displays prominent local convex properties in the vicinity of the actual value. However, the objective function does not exhibit global convex properties, which implies that it possesses multiple local maximums and the conventional local grid search approach might be insufficient and infeasible. Specifically, each parameter shows varying degrees of non-convexity at a global level. The rotation-related parameters, such as yaw, pitch, and roll angles, tend to exhibit a greater degree of convexity than that of translation-related parameters, including x, y, and z translations. This can be attributed, in part, to the fact that rotation can result in a more pronounced alteration of the visual features within the field of view compared to translation alone. For instance, a camera that is translated along a roadway for a limited distance may capture nearly identical visual features within its field of view, whereas a rotational adjustment can induce a more noticeable transformation of the scene.

To investigate the effectiveness of the proposed multi-level optimization approach, we conducted a quantitative analysis of the proposed approach by comparing it with the method proposed in [19]. It should be noted that the latter method employs a distinct data processing technique and objective function as compared to the approach proposed in this study. For the sake of comparison, we employed the same data processing technique and objective function as the one utilized in this paper. We perturbed the ground truth parameters and utilized them as initial values, after which we applied different optimization methods to obtain the calibration parameters. Subsequently, we evaluated the disparities between the optimized parameters and the ground truth parameters.

In this study, the primary assessment metric employed is the Mean Absolute Error (MAE), which provides a clear indication of the disparities between the calibration outcomes and the corresponding ground truth values concerning rotation angles, encompassing yaw, pitch, and roll, as well as translation distances, encompassing x, y, and z translations. Moreover, the standard deviation values of the aforementioned parameters are also calculated to effectively demonstrate the precision of the methods used in the calibration process.

To validate the effectiveness of the proposed approach when faced with poor initial calibration parameters, we utilized uniform random sampling to generate perturbations within a range of [−10°, 10°] and [−1.0 m, 1.0 m], similar to the range in [39]. This range represents a state of poor calibration parameters, which we deemed crucial to the comprehensive assessment of the approach. In each frame, 10 samples were taken. The effectiveness of the approach was assessed by calculating the mean value and standard deviation of the absolute error for yaw, pitch, roll, x, y, and z over all samples in the entire sequence of frames. These metrics were indicative of both the accuracy and precision of the approach. In addition, the average runtime was measured as a means to compute the calibration cost, thereby enabling the assessment of the approaches’ efficiency.

The Levinson method [19] was employed with two different parameter configurations: one with a search radius of 1 (denoted as Lev-1) and the other with a search radius of 2 (denoted as Lev-2). The rotation and translation step sizes for both configurations were set at (0.125° and 5 cm), respectively. These configurations were set up to investigate the impact of different search ranges on calibration accuracy and runtime, and to serve as control groups for the proposed approaches. The proposed approach utilized a desired search range R of (1°, 40 cm), a desired search step size $\tilde{s}$ of (0.125°, 5 cm), a search radius $r_{0}$ of 1, and a step size decrease factor K of 2, where the search step size is reduced to half of the previous search step size at each level. These numerical values were chosen based on current methods [19,39,40,42], and the proposed method configuration is denoted as Pro-1. To evaluate the impact of the enhanced objective definition via suppression of identical pixel contributions, the identical pixel suppression was turned off for a second configuration, denoted as Pro-2. The results of these configurations are presented in detail in Table 3.

Comparing the first two columns of the results, we observe that increasing the search radius can significantly enhance the calibration performance, especially when the initial calibration parameters are poor. However, this comes at the cost of significantly longer average runtimes, making larger search radii impractical due to the computational overhead. The third column demonstrates that the proposed approach in this study can greatly improve optimization performance while maintaining a reasonable computational cost. Comparing the last two columns shows that the identical pixel suppression mechanism helps to achieve a more accurate and precise calibration result.

**Figure 8:**
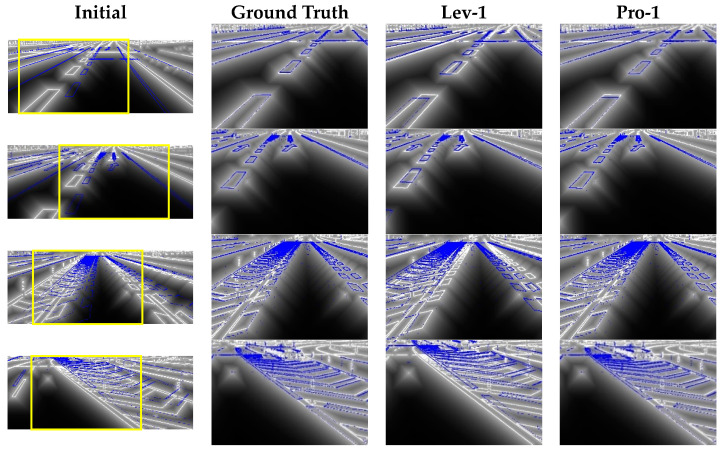
Results of calibration on different frames. The results presented in the latter three columns correspond to the specific region delineated by the yellow boxes in the initial image, in order to enhance visual presentation.

To provide a visual representation of the proposed approach, a set of sample frames were selected and analyzed using both the Levinson method [19] and the proposed method. The calibration results obtained from each method are presented in Figure 8 for comparison. It is important to note that the Levinson method utilized a search radius of 1, ensuring that the runtime of both methods was comparable and that the calibration performance could be evaluated fairly.

The first column in the figure displays the initial overlap of the point cloud on the camera image, which indicates a significant misalignment between these two data sources. The second column shows the overlap achieved by utilizing ground truth calibration parameters, in which a perfect alignment between the two data sources is achieved. The last two columns demonstrate the calibration results obtained by applying the Lev-1 configuration and the proposed method presented in this paper, respectively. The outcomes clearly indicate that the proposed method resulted in a more accurate calibration result.

### 4.3. Ablation Study

The proposed calibration approach incorporates several hyperparameters that require ad hoc determination. In order to assess the significance of various components within the calibration approach, we systematically varied these components and measured the resulting impact on performance. The findings of these experiments are presented in Table 4.

In Table 4 row A, the increase in the search radius $r_{0}$ positively influences both the accuracy and precision of the calibration; however, this improvement is accompanied by a substantial increase in calibration cost, rendering the search radius $r_{0}\;$the parameter with the most substantial influence on the calibration cost. Although a larger search radius expands the field of view during the search process, ensuring a more robust exploration, it is generally recommended to utilize a relatively smaller value to mitigate the associated calibration cost burden. In row B, we investigate the effect of adjusting the desired search step size $\tilde{s}$ for rotation and translation. Generally, a smaller desired search step size leads to more accurate and precise calibration results, albeit at the expense of longer execution time and higher calibration costs. We posit that this is due to the finer granularity of the search process, which enhances the quality of the solution. Nevertheless, the increase in search levels within the multi-level search process prolongs the overall search time. In practical scenarios, we recommend adapting the desired search step size based on the specific calibration requirements. This approach ensures that the calibration process is tailored to meet the desired level of accuracy and precision, thereby optimizing the calibration effectiveness in real-world applications.

In Table 4 row C, we further investigated the impact of the step size decrease factor K on the calibration process. Our observations indicate that a larger value of K generally compromises the quality of calibration while offering the advantage of reduced calibration cost. This phenomenon can be attributed to the fact that a larger K value accelerates the focus on a smaller search area, potentially overlooking certain regions that may contain superior solutions. Simultaneously, the calibration cost decreases due to the expedited convergence of the calibration process. In row D, we conducted an experiment by disabling the suppression of identical pixel contributions, resulting in the improved effectiveness of the calibration approach, as anticipated.

## 5. Limitations and Future Works

The LiDAR utilized in this study pertains to a 2D LiDAR commonly employed in data collection vehicles. However, it is important to note that in the context of autonomous driving vehicles, 3D LiDAR sensors are typically utilized [59]. Present-day 3D LiDAR devices are characterized by sparse point distributions, resulting in relatively low data density compared to camera images. This discrepancy presents challenges when comparing and associating the two data modalities. Moreover, the edge extraction technique presented in this paper may not be directly applicable when the point density is insufficient. Nevertheless, alternative feature extraction techniques [23,26,29,30] that account for the characteristics of low-density point clouds can be employed to address this limitation. Furthermore, as LiDAR sensors continue to advance in terms of capabilities [60], with the emergence of FLASH LiDAR capable of capturing point densities comparable to cameras, the proposed method holds promise for its effective application in the future.

The camera model employed in this study is based on the conventional perspective camera model. However, it is important to acknowledge that wide-angle/fisheye lenses are extensively utilized in real-world applications, particularly in autonomous vehicles, due to their ability to capture wide fields of view, including blind spots [61]. These lenses introduce various forms of visual distortion, with radial distortion being the most prominent geometric effect. Other distortions, such as inaccurate center of distortion estimation and uneven illumination, should also be taken into consideration. These distinctive characteristics of wide-angle/fisheye lenses differentiate them from conventional perspective lenses. Nevertheless, various distortion correction techniques, such as those for radial distortion correction [62,63], center of distortion [64], tangential distortion [65], and uneven illumination [66], are available to mitigate these distortions. Furthermore, several general camera models and calibration methods were proposed to account for the differences between wide-angle/fisheye lenses and perspective lenses [67,68,69]. By employing appropriate correction methods, a fisheye image can be transformed into an approximation of the desired rectilinear model, which is suitable for numerous applications. Similarly, in [27], an equidistant projection for lens projection and a spherical model for panoramic stitching were utilized, resulting in the successful registration of panoramic/fisheye image sequences and LiDAR points using skyline features. The edge-matching technique presented in this paper primarily relies on the pixel differences between adjacent pixels to extract edges from camera images. This approach is less susceptible to the challenges associated with wide-angle/fisheye lenses. Therefore, we argue that by thoroughly considering the characteristics of wide-angle/fisheye lenses, the edge-matching technique described in this paper can be applied without significant obstacles.

The proposed method in this paper demonstrates the potential for further improvement. The current methodology relies on limited data, specifically dense point clouds, and necessitates accurate extraction of road marking point cloud data. A more universal indicator derived from point cloud data could be explored to correspond with image edges. Furthermore, the multi-level optimization algorithm involves numerous hyperparameters, as discussed in Section 4.3, which are currently set on an ad hoc basis. Future research should focus on developing an automatic approach for determining these hyperparameters.

## 6. Conclusions

Achieving accurate calibration between camera and LiDAR sensors is critical for effective information fusion. However, current data-driven calibration methods are susceptible to poor initial calibration parameters, which can lead to suboptimal performance.

This paper proposes a novel camera–LiDAR calibration general model that offers a comprehensive and systematic framework to achieve optimal calibration results, while abstracting away technical details associated with existing methods, enabling researchers and practitioners to comprehend the underlying principles and mechanisms of data-driven calibration. It also provides an objective function that exploits the unique properties of camera image data and LiDAR point clouds. Notably, the proposed objective function takes poor calibration parameters into account, and although it exhibits global non-convexity, it possesses local convexity properties that enable iterative searches for calibration parameters and the optimization of calibration parameters. Balancing precision, robustness, and efficiency during iterative optimization within a local range is a challenging task, but the proposed multi-level optimization method successfully addresses this issue.

In the case of suboptimal initial calibration settings characterized by rotation angle deviations of up to 10° and translation deviations of up to 1.0m from the ground truth, our proposed method achieves a notable enhancement in calibration accuracy. Specifically, the mean calibration error achieved by our method is 0.3° for rotation and 5 cm for translation. This represents a substantial improvement compared to the performance metrics of existing methods, where rotation error reached 1.3° and translation error amounted to 28 cm. These results demonstrate the efficacy and effectiveness of our method in mitigating the adverse effects of poor initial calibration settings and achieving more accurate calibration estimates. In real-world applications, it should be noted that some hyperparameters can be modified to ensure a better balance between better calibration quality and high efficiency of the process. In particular, the search radius should not be increased unless the desired search step size should be determined according to the practical calibration requirements.

## Figures and Tables

**Table 1:** Classification of camera–LiDAR calibration approaches presented in the literature, dissected into four main categories that are target-based approaches, feature matching based approaches, statistics-based approaches, and deep learning-based approaches.

Category	Subcategory	References
Target-Based	-	[20,21,22]
Feature Matching-Based	Point Features	[23,24,25]
	Line Features	[26,27,28]
	Surface Features	[29]
	Semantic Features	[30]
	3D Structure Features	[31]
Statistics-Based	Reflectivity—Grayscale intensity	[32,47]
	Surface normal—Grayscale intensity	[37,38]
	Gradient magnitude and orientation—Gradient magnitude and orientation	[34]
	3D semantic label—2D semantic label	[48]
Deep Learning-Based	Regression	[39,40,41,42,43]
	Calibration Flow	[44,45]
	Keypoints	[46]

**Table 2:** Summary of related work using the proposed general model.

Method	Data Encoding	Objective Definition	Parameter Optimization
[20]	Predefined calibration board features	Combination of reprojection error and laser to calibration plane errors	Levenberg–Marquardt method
[23]	Harris corner extractor and canny edge extractor	Sum of squared error between points from template matching	Least mean squares optimization with RANSAC
[26]	Skyline pixels extractor	Sum of squared error between points from ICP considering the point normal vectors	Least mean squares optimization with RANSAC
[27]	Skyline pixels extractor	Number of matching skyline pixels	Brute force optimization
[31]	Sparse point clouds using SFM	Reprojection error, point-to-plane error, and the difference between camera pose and the pose given by IMU	Bundle adjustment
[32]	Reflectance of LiDAR data and intensity of camera image	Mutual information between image pixel values and laser reflectance intensity	Barzilai–Borwein steepest gradient ascent algorithm [55]
[30]	Semantic segmentation from camera image	Matching degree of the LiDAR points projected into the feature object region	Non-monotonic subgradient ascent algorithm
[39]	Convolutional neural networks	The difference between the real perturbation and network output perturbation	Stochastic gradient descent

**Table 3:** Quantitative result comparison. The assessment of the calibration approach’s effectiveness involves analyzing the mean value and standard deviation of the absolute error for parameters such as yaw, pitch, roll, x, y, and z. The evaluation of efficiency entails quantifying the calibration cost, which is determined by measuring the average runtime of the calibration process.

Configurations		Lev-1	Lev-2	Pro-1	Pro-2
Mean (°)	Yaw	1.2128	0.1732	0.2114	0.3432
	Pitch	1.4432	0.2973	0.3254	0.3965
	Roll	1.1283	0.3343	0.3863	0.4230
	X	0.3453	0.0328	0.0437	0.1032
	Y	0.2476	0.0612	0.0673	0.1187
	Z	0.2309	0.0419	0.0441	0.0872
Standard Deviation (m)	Yaw	0.8635	0.1682	0.2728	0.2983
	Pitch	0.9163	0.2137	0.3146	0.3879
	Roll	1.2734	0.2514	0.4829	0.5654
	X	0.2903	0.0681	0.0900	0.1019
	Y	0.2829	0.0611	0.0735	0.0716
	Z	0.3038	0.0358	0.0838	0.1012
Calibration Cost (TFLOPs) ^1^		7.931	61.314	10.680	10.932

^1^ We used values of 5.99 TFLOPS for RTX 2080 Super with Max-Q Design GPU.

**Table 4:** Variations of several hyper-parameters of the proposed approach. Unlisted values are identical to those of the base settings. MR represents the mean value of the absolute error pertaining to rotation angles, namely yaw, pitch, and roll. Similarly, MT denotes the mean value of the absolute error associated with positional coordinates, specifically x, y, and z. DR and DT stand for the corresponding standard deviations of the aforementioned errors. Furthermore, CC represents the calibration cost, which is quantified by measuring the average duration of the calibration process.

	$r_{0}$	$\tilde{s}$	K	MR (°)	MT (m)	DR (°)	DT (m)	CC (TFLOPs) ^1^
base	1	(0.125°, 5 cm)	2	**0.3077 ^2^**	**0.0517**	**0.3568**	**0.0824**	**10.680**
A	2			0.2973	0.0508	0.3013	0.0733	97.382
	3			0.2952	0.0491	0.2550	0.0498	5523.103
B		(0.0625°, 2.5 cm)		0.2846	0.0477	0.3032	0.0605	13.436
		(0.25°, 10 cm)		0.3212	0.0659	0.3710	0.0831	9.452
		(0.5°, 20 cm)		0.4134	0.0915	0.4334	0.1016	8.170
C			1.5	0.3024	0.0467	0.3030	0.0536	13.939
			1.75	0.3021	0.0489	0.3378	0.0512	11.884
			2.5	0.3324	0.0619	0.3984	0.0963	9.213
			3	0.3712	0.0987	0.4770	0.1289	7.194
D	without suppression of identical pixel contributions			0.3876	0.1030	0.4172	0.0916	10.932

^1^ We used values of 5.99 TFLOPS for RTX 2080 Super with Max-Q Design GPU. ^2^ The metrics associated with the base settings are highlighted in bold for more convenient comparisons with other variations.

## Data Availability

The data presented in this study are available on request from the corresponding author.
